# The vasoactive-age adjusted sepsis-induced coagulopathy score predicts 28-day new-onset multiple organ dysfunction syndrome in patients with sepsis: a single-centre retrospective cohort study

**DOI:** 10.3389/fmed.2026.1874204

**Published:** 2026-07-06

**Authors:** Ke-jun Cao, Jia-qi Ge, Hong-zhi Fang

**Affiliations:** 1 Department of Critical Care Medicine, The Affiliated Hospital of Jiangnan University, Wuxi, Jiangsu, China; 2 Emergency Department, Jiangnan University Medical Center (JUMC), Wuxi, Jiangsu, China

**Keywords:** multiple organ dysfunction syndrome, nomogram, prognosis, risk stratification, sepsis, sepsis-induced coagulopathy

## Abstract

**Background:**

Early identification of sepsis patients at high risk of multiple organ dysfunction syndrome (MODS) remains challenging. The Sepsis-Induced Coagulopathy (SIC) score does not incorporate age or vasopressor use, both strongly associated with organ failure progression. The Vasoactive-Age adjusted SIC (VAS) score integrates these dimensions, but its predictive value for new-onset MODS has not been systematically evaluated.

**Methods:**

This single-centre retrospective cohort study enrolled 495 adult sepsis patients (Sepsis-3.0 criteria) admitted between January 2019 and June 2023. Baseline VAS, SIC, Sequential Organ Failure Assessment (SOFA), 24-h worst SOFA (SOFA-2), Acute Physiology and Chronic Health Evaluation II (APACHE II), and quick SOFA (qSOFA) scores were calculated. The primary outcome was 28-day new-onset MODS. Predictive performance was compared using the area under the receiver operating characteristic curve (AUC) and the DeLong method. Cumulative incidence was estimated with Fine–Gray competing-risk models. Independent predictors were identified by multivariable Cox regression. Net reclassification improvement (NRI), integrated discrimination improvement (IDI), and decision curve analysis quantified incremental value. A nomogram was internally validated by 1,000-bootstrap resampling.

**Results:**

New-onset MODS occurred in 148 patients (29.9%). The VAS score yielded an AUC of 0.777 [95% confidence interval (CI) 0.755–0.841], significantly higher than SIC (0.708, *p* < 0.001) and qSOFA (0.671, *p* < 0.001), and comparable to SOFA (0.757, *p* = 0.083). The optimal cut-off of VAS ≥ 4 yielded 76.4% sensitivity and 73.2% specificity. After multivariable adjustment, VAS ≥ 4 remained independently predictive of MODS [hazard ratio (HR) 3.42, 95% CI 2.31–5.07, *p* < 0.001], with significant interactions for age (*p* = 0.024), septic shock (*p* = 0.038), and SIC status (*p* = 0.041). Adding lactate, procalcitonin, and N-terminal pro-B-type natriuretic peptide increased the AUC to 0.853 (NRI = 0.124, *p* = 0.012; IDI = 0.086, *p* = 0.021). A dynamic VAS increase (ΔVAS>0) over 48 h was independently associated with a 4.78-fold higher MODS risk (*p* < 0.001). The internally validated nomogram achieved a corrected C-index of 0.821.

**Conclusion:**

The VAS score is a simple bedside tool that independently predicts 28-day new-onset MODS in sepsis, outperforming the SIC score and matching SOFA with fewer parameters. Combination with circulating biomarkers and dynamic ΔVAS monitoring provides additional prognostic value and supports its integration into early sepsis risk stratification.

## Introduction

Sepsis—life-threatening organ dysfunction caused by a dysregulated host response to infection—remains one of the foremost challenges in critical care medicine. The Global Burden of Disease Study estimated 48.9 million incident sepsis cases and 11 million sepsis-related deaths in 2017, accounting for nearly 20% of all global deaths (1). Multiple organ dysfunction syndrome (MODS) represents the most severe consequence of sepsis, simultaneously involving the respiratory, circulatory, coagulation, hepatic, renal, and central nervous systems, with reported mortality of 40–60% even in well-resourced intensive care units (ICUs) (2, 3). Accurate early recognition of patients at high risk of MODS is therefore central to optimising treatment timing and improving outcomes.

Current risk-stratification tools for sepsis include the Sequential Organ Failure Assessment (SOFA), Acute Physiology and Chronic Health Evaluation II (APACHE II), and quick SOFA (qSOFA) scores (4). The SOFA score is the reference standard for organ-dysfunction quantification but requires a comprehensive set of laboratory and haemodynamic parameters that are often unavailable in early emergency-department evaluation (5). APACHE II reliably predicts ICU mortality but uses the worst values within the first 24 h after admission, which precludes real-time decision-making (6). The qSOFA score is bedside-friendly but lacks sufficient sensitivity to detect patients with progressive organ failure (7). None of these scores incorporates the coagulation system, despite the fact that sepsis-induced coagulopathy (SIC) is a key precursor of organ dysfunction whose early identification has major clinical implications (8).

The SIC score, proposed by the International Society on Thrombosis and Haemostasis (ISTH) in 2017, integrates the prothrombin time (PT) ratio, platelet count, and SOFA score, and has been validated as an early indicator of coagulation derangement and adverse outcomes in sepsis (9, 10). Compared with overt disseminated intravascular coagulation (DIC), the SIC score detects coagulation abnormalities earlier and may inform the timing of anticoagulation (11). Two intrinsic limitations remain. First, the SIC score does not incorporate age, although age is an important independent determinant of organ reserve and immune competence in sepsis: elderly patients—because of immunosenescence and reduced organ reserve—develop MODS at higher rates than younger patients with comparable disease severity (12, 13). Second, the SIC score does not capture the magnitude of circulatory failure, even though vasopressor use is a defining element of septic shock and is closely linked to tissue hypoperfusion and downstream organ injury (14, 15); explicit incorporation of vasopressor status therefore offers an objective, immediately available proxy of haemodynamic decompensation that complements coagulation-centred information. These gaps create an information blind spot for integrative MODS prediction.

The Vasoactive-Age adjusted SIC (VAS) score adds two dimensions—vasopressor use and age—to the original SIC score and is theoretically positioned to overcome these limitations. However, independent validation remains scarce. Its predictive performance for new-onset MODS, the optimal clinical cut-off, its relative performance against established scores, the incremental value of combining it with circulating biomarkers such as lactate, procalcitonin (PCT), and N-terminal pro-B-type natriuretic peptide (NT-proBNP), the prognostic significance of dynamic changes (ΔVAS), and its applicability across clinical subgroups have not been systematically examined (16, 17).

In this single-centre retrospective cohort study, we evaluated the VAS score for prediction of 28-day new-onset MODS in patients with sepsis, compared its performance with that of the SIC, SOFA, and APACHE II scores, quantified the incremental value of biomarker combinations, examined the prognostic implications of dynamic ΔVAS, characterised performance across pre-specified subgroups, and developed an individualised risk-prediction nomogram.

## Methods

### Study design and population

This single-centre retrospective cohort study consecutively enrolled adult patients with sepsis (Sepsis-3.0 criteria—suspected or confirmed infection plus an acute increase in SOFA score of ≥2 points from baseline) (4) admitted to the emergency department or ICU of our hospital between January 2019 and June 2023. The protocol was approved by the institutional ethics committee of the Affiliated Hospital of Jiangnan University which waived informed consent.

Inclusion criteria: (1) age ≥18 years; (2) complete blood count, coagulation panel, arterial blood gas analysis, lactate, and biochemistry obtained within 6 h of admission; (3) hospital stay ≥24 h; (4) absence of MODS at baseline (to enable evaluation of “new-onset” organ dysfunction); (5) data sufficient to calculate the VAS, SIC, SOFA, and APACHE II scores; (6) availability of 28-day follow-up.

Exclusion criteria: (1) ≥ 2 organ-system SOFA sub-scores ≥3 at admission (i.e., baseline MODS); (2) hospital-acquired infection; (3) regular pre-admission use of warfarin, heparin, or direct oral anticoagulants; (4) prior haematological disease or primary thrombocytopenia; (5) end-stage malignancy, brain death, or treatment limitation at admission; (6) pregnancy or lactation; (7) missing key variables precluding score calculation, or ≥20% missing 28-day outcome data.

### VAS score and comparator scoring systems

The VAS score (range 0–9) extends the SIC score by integrating two correction modules:

Coagulation module – PT ratio: <1.2, 0 points; 1.2–<1.4, 1 point; ≥1.4, 2 points. Coagulation module – platelet count: ≥150 × 10^9^/L, 0 points; 100–<150 × 10^9^/L, 1 point; <100 × 10^9^/L, 2 points. Organ-function module – SOFA: SOFA <2, 0 points; SOFA ≥2, 2 points. Vasopressor module: any vasopressor use at admission (norepinephrine, epinephrine, dopamine, or vasopressin), 1 point; otherwise 0 points. Age module: <60 years, 0 points; 60–74 years, 1 point; ≥75 years, 2 points. Vasopressor use was incorporated because it is an objective bedside marker of refractory circulatory failure persisting despite adequate fluid resuscitation, directly reflects the severity of septic-shock-related hypoperfusion, and represents an independent dimension of organ injury not captured by coagulation parameters or the binary SOFA component alone.

Higher scores reflect greater risk of organ deterioration and MODS; the optimal cut-off was determined by the Youden index. SOFA was recorded at admission (T₀) and at 24 h (T₂₄) using the worst values; as a sensitivity-analysis comparator, SOFA-2 was defined as the worst SOFA score obtained within the first 24 h after admission; SIC was scored from PT ratio, platelet count, and SOFA (≥4 considered SIC-positive); APACHE II used the worst physiological parameters within 24 h; qSOFA was based on altered mentation, respiratory rate ≥22/min, and systolic blood pressure ≤100 mmHg at T₀. The dynamic change was defined as ΔVAS = VAS at T₄₈ − VAS at T₀. Scores were calculated by trained intensive-care researchers using a standardised manual; inter-rater agreement was targeted at *κ* > 0.85.

### Outcome definitions

The primary outcome was 28-day new-onset MODS, defined as the occurrence at any point during 28-day follow-up of simultaneous SOFA sub-scores ≥3 in ≥2 organ systems (respiratory, coagulation, hepatic, circulatory, central nervous, or renal) in patients without MODS at baseline. Two independent investigators adjudicated outcomes; discrepancies were resolved by consensus or a third adjudicator.

Secondary outcomes were: (1) overt DIC (ISTH 2001 criteria, score ≥5); (2) acute kidney injury (AKI; Kidney Disease: Improving Global Outcomes [KDIGO] 2012 criteria); (3) new initiation of invasive mechanical ventilation; (4) new initiation of continuous renal replacement therapy (CRRT); (5) septic shock (Sepsis-3 definition); (6) a composite major adverse clinical events (MACE) endpoint—occurrence of any of new-onset MODS, new invasive mechanical ventilation, new CRRT, new overt DIC, or escalation from a general ward to the ICU; and (7) 30-day readmission.

### Data collection

Baseline data included demographics, the Charlson Comorbidity Index (CCI), source of infection, vital signs at admission, and a complete laboratory panel (haematology, coagulation, blood gas, biochemistry, and inflammatory markers). Inflammatory and cardiac biomarkers comprised PCT, C-reactive protein (CRP), and NT-proBNP; PCT was log₁₀-transformed to address skewness. Treatment data included time from admission to first antibiotic dose (h), 6-h fluid resuscitation volume (mL), and vasopressor use. Twenty-eight-day outcomes were ascertained through outpatient records and structured telephone follow-up; the target loss-to-follow-up rate was <5%.

### Statistical analysis

Continuous variables were summarised as mean ± standard deviation or median [interquartile range (IQR)] and compared using Student’s *t*-test or the Mann–Whitney U-test. Categorical variables were summarised as *n* (%) and compared using the *χ*^2^ test or Fisher’s exact test. Missing data were <5% for every variable (per-variable rates in Supplementary Table S5); missing continuous values were addressed by multiple imputation by chained equations (MICE, *m* = 5), and sensitivity analyses restricted to complete cases yielded qualitatively consistent results.

Discrimination and calibration. Receiver operating characteristic (ROC) curves were constructed and the area under the curve (AUC), optimal cut-off, sensitivity, specificity, positive and negative predictive values (PPV/NPV), and positive and negative likelihood ratios (+LR/−LR) were calculated. Pairwise AUCs were compared using the DeLong method. Calibration was evaluated by the Hosmer-Lemeshow test and calibration curves; clinical utility by decision curve analysis (DCA).

Incremental value. Using SOFA as the base model, VAS modules and biomarkers were sequentially added; net reclassification improvement (NRI) and integrated discrimination improvement (IDI) quantified incremental contributions.

Time to first MODS was the event of interest; patients without MODS were censored at day 28. Cumulative incidence was estimated using Fine-Gray competing-risk models with death as the competing event, and Cox proportional-hazards regression identified independent predictors. The primary multivariable model (Table 1) included VAS category plus age, Charlson Comorbidity Index (CCI), time to antibiotic initiation, 6-h fluid resuscitation volume, lactate, and log₁₀(PCT) (7 variables; events-per-variable ratio = 21.1). Subgroup analyses (Table 2; Supplementary Table S4) used a reduced set—age, CCI, source of infection, time to antibiotic initiation, and 6-h fluid volume—to preserve statistical power within strata. The dynamic-trajectory model (Supplementary Table S2) additionally adjusted for baseline VAS to isolate the prognostic information carried by ΔVAS beyond the starting score.

**Table 1:** Cox proportional hazards regression analysis of VAS score for predicting 28-day new-onset MODS.

Variable	Univariable HR (95% CI)	*p* value	Multivariable HR (95% CI)ᵃ	*p* value
VAS score, per 1-point increase	1.42 (1.32–1.53)	<0.001	1.36 (1.25–1.48)	<0.001
VAS category				
Low-risk (VAS < 4), *n* = 289	1.00 (ref.)	—	1.00 (ref.)	—
High-risk (VAS ≥ 4), *n* = 206	4.18 (2.92–5.99)	<0.001	3.42 (2.31–5.07)	<0.001
Age, per 10-year increase	1.32 (1.18–1.48)	<0.001	1.18 (1.04–1.34)	0.012
Male sex	1.16 (0.84–1.61)	0.371	Not included	—
Charlson index, per 1-point increase	1.18 (1.10–1.27)	<0.001	1.10 (1.02–1.19)	0.018
Pulmonary infection (vs. other)	1.21 (0.87–1.69)	0.250	Not included	—
Antibiotic delay, per 1 h	1.06 (1.02–1.10)	0.003	1.04 (1.00–1.08)	0.046
Fluid resuscitation, per 500 mL	1.21 (1.09–1.34)	<0.001	1.13 (1.02–1.26)	0.024
Lactate, per 1 mmol/L increase	1.32 (1.24–1.41)	<0.001	1.18 (1.10–1.27)	<0.001
log(PCT), per 1-unit increase	1.41 (1.24–1.61)	<0.001	1.16 (1.00–1.34)	0.052

ᵃPrimary multivariable Cox model. Adjustment variables: age, Charlson Comorbidity Index, time to antibiotic initiation, 6-h fluid resuscitation volume, blood lactate, log₁₀(PCT), and VAS category (7 variables; events-per-variable ratio = 148/7 = 21.1). See Methods for rationale of covariate selection.

**Table 2:** Pre-specified subgroup analysis of VAS score for predicting 28-day new-onset MODS.

Subgroup	*n*	Events, *n*	HR (95% CI)ᵃ	AUC (95% CI)	P-int
Age					0.024
<60 years	155	27	2.18 (1.04–4.55)	0.726 (0.638–0.814)	
60–74 years	192	58	3.15 (1.92–5.16)	0.781 (0.713–0.849)	
≥75 years	148	63	4.86 (2.84–8.32)	0.832 (0.762–0.902)	
Source of infection					0.412
Pulmonary	225	73	3.28 (2.05–5.25)	0.794 (0.738–0.850)	
Abdominal	95	28	3.95 (1.78–8.76)	0.821 (0.732–0.910)	
Urinary	99	21	2.68 (1.10–6.52)	0.762 (0.658–0.866)	
Other	76	26	3.04 (1.44–6.40)	0.778 (0.665–0.891)	
Septic shock					0.038
No	356	78	2.84 (1.82–4.43)	0.752 (0.692–0.812)	
Yes	139	70	4.62 (2.66–8.02)	0.842 (0.778–0.906)	
CKD					0.156
No	406	110	3.16 (2.13–4.69)	0.781 (0.733–0.829)	
Yes	89	38	4.42 (2.04–9.58)	0.838 (0.749–0.927)	
Diabetes mellitus					0.682
No	347	96	3.32 (2.18–5.06)	0.794 (0.745–0.843)	
Yes	148	52	3.68 (1.94–6.98)	0.806 (0.728–0.884)	
SIC status					0.041
Negative (SIC <4)	298	56	2.46 (1.45–4.18)	0.728 (0.658–0.798)	
Positive (SIC ≥4)	197	92	4.18 (2.46–7.10)	0.806 (0.742–0.870)	
Sex					0.583
Male	304	95	3.42 (2.18–5.36)	0.802 (0.749–0.855)	
Female	191	53	3.18 (1.78–5.69)	0.788 (0.718–0.858)	

ᵃHR represents high-risk (VAS ≥4) vs. low-risk (VAS <4) within each subgroup, adjusted for age, Charlson Comorbidity Index, source of infection, time to antibiotic initiation, and 6-h fluid resuscitation volume. A reduced covariate set (relative to the primary model in Table 1) was applied to maintain an adequate events-per-variable ratio within smaller strata.

Nomogram. A multivariable logistic regression was used to build an individualised nomogram. Internal validation employed 1,000-bootstrap resampling, reporting the corrected concordance index (C-index), calibration intercept, and slope.

All analyses were conducted in SPSS 26.0 and R (≥4.3.0; pROC, survival, survminer, cmprsk, rmda, ggplot2, forestplot). Two-sided *p* < 0.05 was considered statistically significant.

## Results

### Patient characteristics

Of 583 adult patients with suspected or confirmed sepsis screened during the study period, 495 met the eligibility criteria and were included in the analytic cohort; 28-day follow-up was complete for 99.2%. Exclusions comprised baseline MODS (n = 28), hospital-acquired infection (n = 18), regular pre-admission anticoagulant use (n = 12), prior haematological disease or primary thrombocytopenia (n = 7), end-stage malignancy/brain death/treatment limitation (n = 10), pregnancy or lactation (n = 3), and missing key variables or ≥20% missing outcome data (n = 10). Median age was 66 years (IQR 54–77), 304 (61.4%) were male, and pulmonary infection was the most common source (45.5%). New-onset MODS occurred in 148 patients (29.9%). Compared with the no-MODS group, the new-onset MODS group was older, had a higher CCI, more frequent CKD and chronic heart failure, higher admission lactate, PCT, creatinine, and vasopressor use, lower platelet count and arterial oxygen partial pressure to fractional inspired oxygen ratio (PaO₂/FiO₂), and significantly higher baseline VAS, SOFA, and APACHE II scores (all *p* < 0.001). Sex, body mass index, and infection-source distribution did not differ between groups (Table 3).

**Table 3:** Baseline characteristics of patients stratified by 28-day new-onset MODS.

Variable	All patients (*N* = 495)	New-onset MODS (*n* = 148)	No MODS (*n* = 347)	*p* value
Demographics				
Age, years, *M* [IQR]	66 [54, 77]	72 [60, 80]	64 [52, 75]	<0.001
Male, n (%)	304 (61.4)	95 (64.2)	209 (60.2)	0.421
BMI, kg/m^2^, mean±SD	22.9 ± 3.5	23.1 ± 3.6	22.8 ± 3.4	0.378
Comorbidities, *n* (%)				
Hypertension	266 (53.7)	88 (59.5)	178 (51.3)	0.094
Diabetes mellitus	148 (29.9)	52 (35.1)	96 (27.7)	0.097
COPD	88 (17.8)	32 (21.6)	56 (16.1)	0.139
CKD	89 (18.0)	38 (25.7)	51 (14.7)	0.003
Chronic heart failure	71 (14.3)	30 (20.3)	41 (11.8)	0.012
Malignancy (non-terminal)	63 (12.7)	24 (16.2)	39 (11.2)	0.124
Charlson comorbidity index, M [IQR]	3 [2, 5]	4 [3, 6]	3 [2, 5]	<0.001
Source of infection, *n* (%)				0.262
Pulmonary	225 (45.5)	73 (49.3)	152 (43.8)	
Abdominal	95 (19.2)	28 (18.9)	67 (19.3)	
Urinary	99 (20.0)	21 (14.2)	78 (22.5)	
Bloodstream	42 (8.5)	14 (9.5)	28 (8.1)	
Other/unknown	34 (6.9)	12 (8.1)	22 (6.3)	
Baseline scores, M [IQR]				
VAS score	3 [2, 5]	5 [4, 6]	2 [1, 3]	<0.001
SIC score	2 [1, 4]	4 [3, 5]	2 [1, 3]	<0.001
SOFA score	5 [3, 7]	7 [5, 9]	4 [2, 5]	<0.001
APACHE II score	14 [10, 19]	19 [15, 23]	13 [9, 17]	<0.001
qSOFA score	1 [1, 2]	2 [1, 2]	1 [0, 2]	<0.001
Laboratory values, M [IQR]				
WBC, ×10^9^/L	12.6 [8.4, 17.2]	14.2 [9.6, 19.8]	11.8 [7.9, 16.4]	0.002
Platelet, ×10^9^/L	178 [118, 236]	132 [86, 198]	198 [142, 256]	<0.001
PT ratio	1.18 [1.08, 1.32]	1.32 [1.18, 1.51]	1.14 [1.05, 1.26]	<0.001
Lactate, mmol/L	2.1 [1.4, 3.4]	3.2 [1.9, 5.1]	1.8 [1.2, 2.7]	<0.001
PCT, ng/mL	4.2 [1.1, 14.6]	8.6 [2.4, 26.3]	2.8 [0.7, 9.4]	<0.001
CRP, mg/L	126 [64, 184]	142 [78, 203]	118 [56, 178]	0.018
Creatinine, μmol/L	108 [80, 156]	142 [98, 218]	92 [72, 128]	<0.001
PaO₂/FiO₂	285 [218, 348]	218 [156, 298]	312 [245, 368]	<0.001
Treatment interventions				
Vasopressor use, *n* (%)	179 (36.2)	92 (62.2)	87 (25.1)	<0.001
Fluid resuscitation within 6 h, mL	2,500 [2000, 3,100]	2,800 [2,200, 3,500]	2,400 [1800, 2,900]	<0.001
Time to antibiotic initiation, h	2.0 [1.1, 3.6]	2.4 [1.3, 4.6]	1.8 [0.9, 3.2]	0.005

M [IQR], median [interquartile range]; mean±SD, mean ± standard deviation; *n* (%), frequency (percentage). Comparisons: continuous variables by independent t-test or Mann–Whitney U test; categorical variables by *χ*^2^ test or Fisher exact test. BMI, body mass index; COPD, chronic obstructive pulmonary disease; CKD, chronic kidney disease; WBC, white blood cell; PT, prothrombin time; PCT, procalcitonin; CRP, C-reactive protein.

### Predictive performance of the VAS score and comparison with other scores

**Figure 1:**
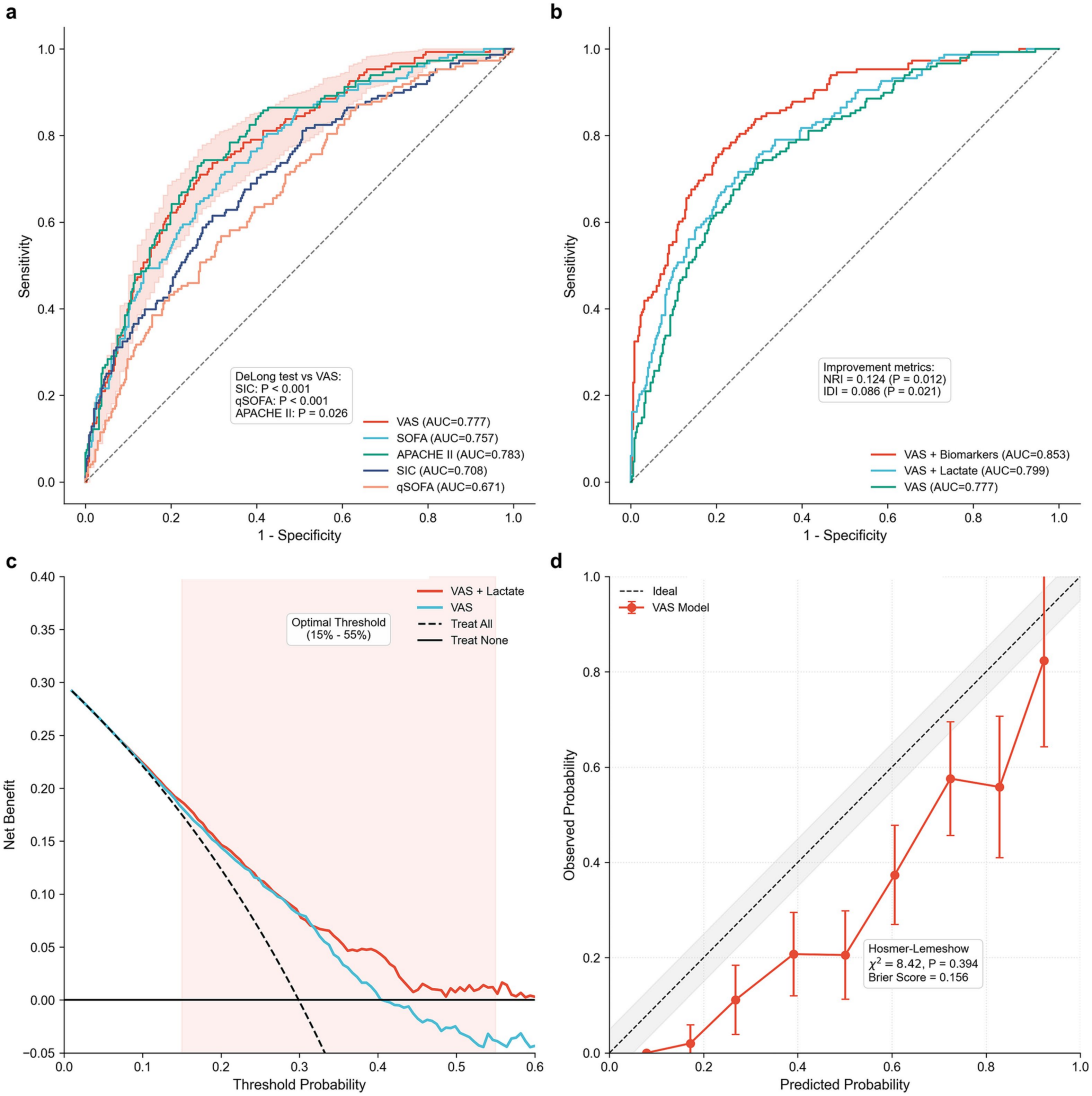
Predictive performance of the VAS score for 28-day new-onset MODS. **(a)** ROC curves for VAS (red, AUC = 0.777), SOFA (light blue, AUC = 0.757), APACHE II (dark green, AUC = 0.783), SIC (dark blue, AUC = 0.708), and qSOFA (light orange, AUC = 0.671). DeLong test versus VAS: SIC *p* < 0.001, qSOFA *p* < 0.001, APACHE II *p* = 0.026. **(b)** ROC curves for VAS + biomarkers (red, AUC = 0.853), VAS + lactate (light blue, AUC = 0.799), and VAS alone (dark green, AUC = 0.777); improvement metrics: NRI = 0.124 (*p* = 0.012), IDI = 0.086 (*p* = 0.021). **(c)** Decision curve analysis comparing VAS alone (light blue) and VAS + lactate (red); the pink shaded zone (threshold probability 15–55%) indicates the clinical decision range in which both models outperform the “treat-all” and “treat-none” reference strategies. **(d)** Calibration curve of the VAS logistic-regression model: red points, observed versus predicted probability; black dashed line, ideal calibration; grey shading, 95% CI; Hosmer-Lemeshow *χ*^2^ = 8.42, *p* = 0.394; Brier score = 0.156.

The VAS score predicted 28-day new-onset MODS with an AUC of 0.777 (95% CI 0.755–0.841); the optimal cut-off was VAS ≥ 4 (sensitivity 76.4%, specificity 73.2%, PPV 54.8%, NPV 88.2%, +LR 2.85, −LR 0.32). The SIC (AUC = 0.708) and qSOFA (AUC = 0.671) scores were significantly inferior (DeLong *p* < 0.001 for both); APACHE II (AUC = 0.783) differed significantly from VAS (*p* = 0.026); SOFA (AUC = 0.757) did not (*p* = 0.083; Figure 1a; Table 4). In a sensitivity analysis, SOFA-2 yielded an AUC of 0.791 (95% CI 0.748–0.834), modestly higher than baseline SOFA but not statistically different from the VAS score (DeLong *p* = 0.412; Table 4).

**Table 4:** Diagnostic performance of scoring systems for predicting 28-day new-onset MODS (ROC analysis).

Score	AUC (95% CI)	Optimal cutoff	Sensitivity, %	Specificity, %	PPV, %	NPV, %	+LR	-LR	P vs. VAS
VAS score	0.777(0.755–0.841)	≥4	76.4	73.2	54.8	88.2	2.85	0.32	Ref.
SIC score	0.708 (0.638–0.736)	≥4	64.2	65.7	44.4	81.2	1.87	0.55	<0.001
SOFA score	0.757 (0.717–0.807)	≥6	71.6	70.0	50.5	85.4	2.39	0.41	0.083
SOFA-2	0.791 (0.748–0.834)	≥7	74.3	71.5	52.6	86.8	2.61	0.36	0.412
APACHE II	0.783 (0.691–0.815)	≥17	68.9	68.0	48.0	83.6	2.15	0.46	0.026
qSOFA	0.671 (0.589–0.695)	≥2	58.1	64.6	41.2	78.6	1.64	0.65	<0.001

P vs. VAS: pairwise AUC comparison by DeLong method. AUC, area under the receiver operating characteristic curve; CI, confidence interval; PPV, positive predictive value; NPV, negative predictive value; +LR, positive likelihood ratio; −LR, negative likelihood ratio; Ref., reference. SOFA-2, the worst SOFA score obtained within the first 24 h after admission, was included as a sensitivity-analysis comparator.

Using SOFA (AUC = 0.757) as the base model, integration of the full VAS score increased the AUC to 0.777 (NRI = 0.28, IDI = 0.038, both *p* < 0.001). Sequential addition of lactate raised the AUC to 0.799, and lactate + PCT + NT-proBNP to 0.853, with NRI = 0.124 (*p* = 0.012) and IDI = 0.086 (*p* = 0.021) compared with VAS alone (Figure 1b; Table 5). DCA showed sustained net benefit of VAS + lactate over VAS alone and the “treat-all” strategy across threshold probabilities of 15–55% (Figure 1c). The VAS model was well calibrated (Hosmer-Lemeshow *χ*^2^ = 8.42, *p* = 0.394; Brier score = 0.156; Figure 1d).

**Table 5:** Incremental predictive value of VAS score modules and biomarker combinations (SOFA as base model).

Model	AUC (95% CI)	NRIᵃ (95% CI)	NRI P	IDI (95% CI)	IDI P
Model 1: SOFA alone	0.762 (0.717–0.807)	Reference	—	Reference	—
Model 2: SOFA + vasopressor module	0.778 (0.733–0.823)	0.18 (0.06–0.30)	0.004	0.022 (0.008–0.036)	0.002
Model 3: SOFA + age-adjustment module	0.776 (0.731–0.821)	0.16 (0.04–0.28)	0.008	0.018 (0.005–0.031)	0.006
Model 4: Full VAS score	0.777 (0.755–0.841)	0.28 (0.15–0.41)	<0.001	0.038 (0.020–0.056)	<0.001
Model 5: VAS + lactate	0.799 (0.742–0.822)	0.42 (0.28–0.56)	<0.001	0.061 (0.038–0.084)	<0.001
Model 6: VAS + lactate + PCT	0.846 (0.808–0.884)	0.46 (0.32–0.60)	<0.001	0.072 (0.046–0.098)	<0.001
Model 7: VAS + lactate + PCT + NT-proBNP	0.853 (0.815–0.889)	0.48 (0.34–0.62)	<0.001	0.078 (0.050–0.106)	<0.001

𝐼ontinuous NRI. All comparisons referenced to Model 1 (SOFA alone). AUC, area under the receiver operating characteristic curve; NRI, net reclassification improvement; IDI, integrated discrimination improvement; PCT, procalcitonin; NT-proBNP, N-terminal pro-B-type natriuretic peptide.

### Cumulative incidence and dynamic ΔVAS analysis

**Figure 2:**
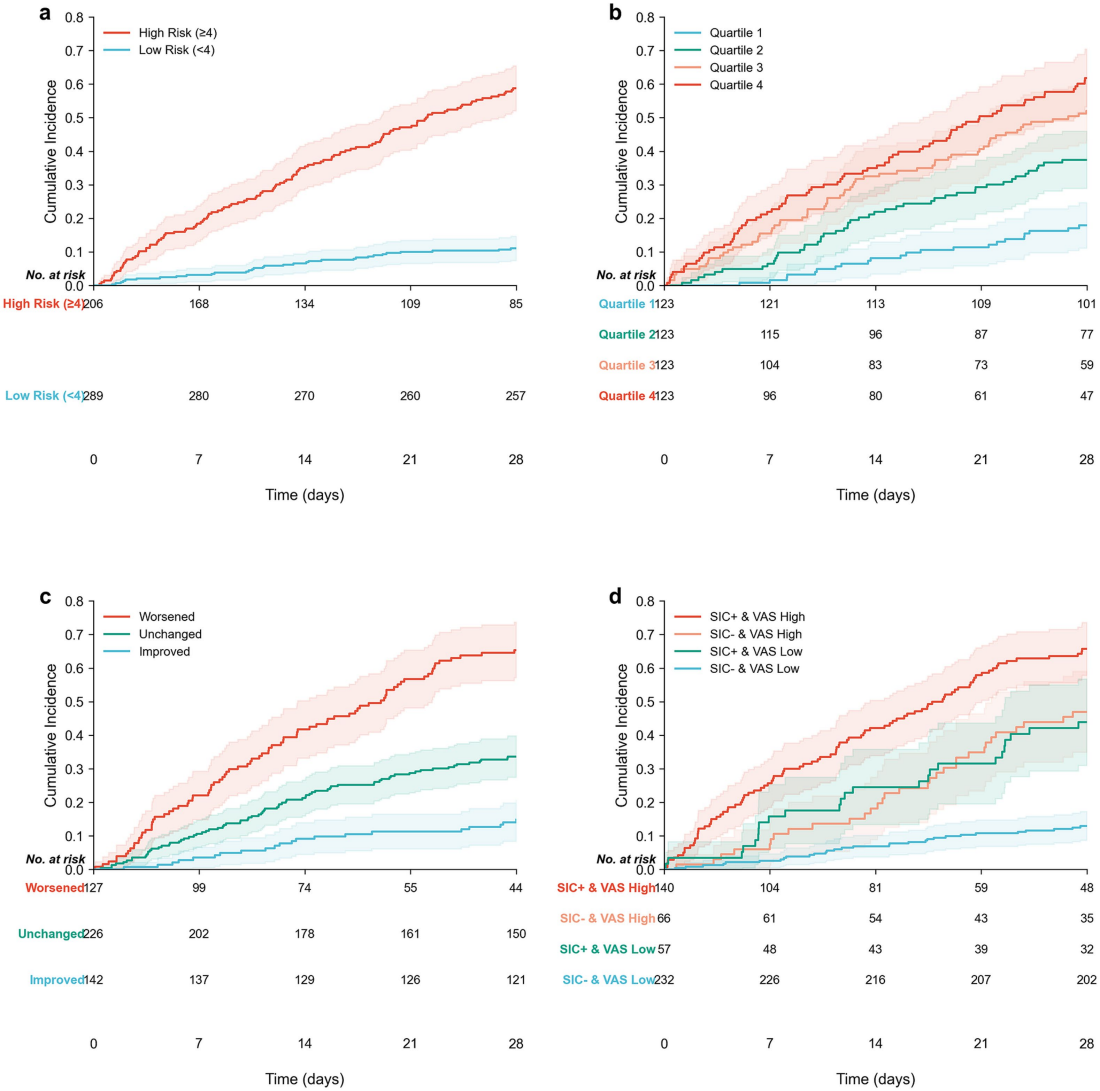
Cumulative incidence curves of 28-day new-onset MODS estimated by Fine-Gray competing-risk models (death as competing event); shading indicates 95% CIs and numbers below indicate at-risk patients. **(a)** High-risk (VAS ≥ 4, *n* = 206, red) versus low-risk (VAS < 4, *n* = 289, blue) groups. **(b)** Cumulative incidence stratified by VAS-score groups defined by quartile cut-points: Q1 (VAS 0–1, *n* = 107), Q2 (VAS 2, *n* = 108), Q3 (VAS 3–4, *n* = 140), and Q4 (VAS ≥ 5, *n* = 140), showing a graded dose–response pattern. **(c)** ΔVAS strata: worsened (>0, *n* = 127, red), unchanged (=0, *n* = 226, dark green), improved (<0, *n* = 142, light blue). **(d)** Combined SIC × VAS stratification: SIC-positive + VAS-high (*n* = 140, red), SIC-negative + VAS-high (*n* = 66, light orange), SIC-positive + VAS-low (*n* = 57, dark green), SIC-negative + VAS-low (*n* = 232, light blue).

The 28-day cumulative incidence of MODS was significantly higher in the high-risk group (VAS ≥ 4, *n* = 206) than in the low-risk group (VAS < 4, *n* = 289) at every follow-up time point (Gray’s test *p* < 0.001; Figure 2a). When stratified by VAS-score groups defined by quartile cut-points (Q1: VAS 0–1, *n* = 107; Q2: VAS 2, *n* = 108; Q3: VAS 3–4, *n* = 140; Q4: VAS ≥ 5, *n* = 140), the cumulative incidence rose monotonically across groups (trend *p* < 0.001; Figure 2b).

By ΔVAS category, 28-day MODS rates were 12.7% (improved, *n* = 142), 28.3% (unchanged, *n* = 226), and 52.0% (worsened, *n* = 127) (trend *p* < 0.001). Multivariable Cox regression confirmed that, compared with the improved group, the unchanged (HR 2.31, 95% CI 1.37–3.89) and worsened (HR 4.78, 95% CI 2.83–8.07) groups had significantly elevated risk (both *p* < 0.01; Figure 2c; Supplementary Table S2).

In the SIC × VAS combined stratification, MODS rates were 9.1% in SIC-negative + VAS-low (reference, *n* = 232), 24.6% in SIC-positive + VAS-low (HR 2.84, *p* = 0.003, *n* = 57), 53.0% in SIC-negative + VAS-high (HR 6.85, *p* < 0.001, *n* = 66), and 55.7% in SIC-positive + VAS-high (HR 7.42, *p* < 0.001, *n* = 140); the trend test was *p* < 0.001 (Figure 2d; Supplementary Table S4).

### Independent predictive value and subgroup analysis

After adjustment for confounders, VAS ≥ 4 was an independent predictor of MODS (HR 3.42, 95% CI 2.31–5.07, *p* < 0.001); each one-point increase in continuous VAS conferred an HR of 1.36 (95% CI 1.25–1.48, *p* < 0.001; Table 1).

**Figure 3:**
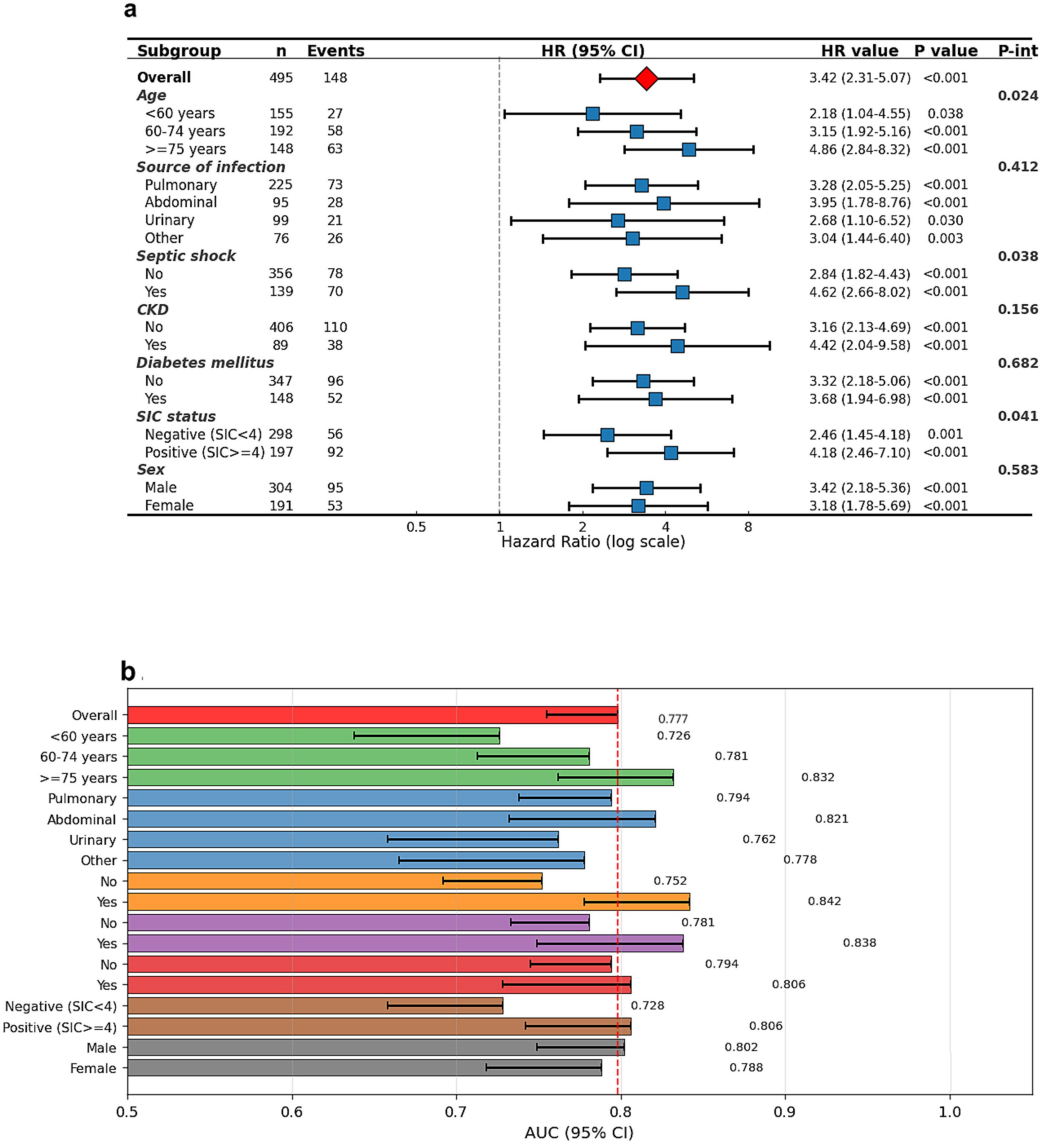
Pre-specified subgroup analyses for prediction of 28-day new-onset MODS. **(a)** Forest plot of VAS-high versus VAS-low hazard ratios. The red diamond is the overall HR (3.42, 95% CI 2.31–5.07, *p* < 0.001); blue squares are subgroup point estimates with 95% CIs. The vertical dashed line denotes HR = 1 (X-axis on log scale). Subgroups: age, source of infection, septic shock, CKD, diabetes mellitus, SIC status, and sex. **(b)** Subgroup AUCs (95% CIs as error bars); the red dashed line marks the overall AUC of 0.777. The age subgroups showed a significant interaction (P-int = 0.024), with the highest AUC in the ≥75-years subgroup (0.832) and the lowest in the <60-years subgroup (0.726).

In all pre-specified subgroups, the VAS-high HR was significantly >1 (Figure 3a; Table 2). Significant interactions were observed for age (P-int = 0.024)—HR was highest in the ≥75 years subgroup (4.86) and lowest in the <60 years subgroup (2.18)—septic shock (P-int = 0.038, HR 4.62 vs. 2.84 in non-shock), and SIC status (P-int = 0.041, HR 4.18 in SIC-positive vs. 2.46 in SIC-negative). No significant interaction was found for source of infection, CKD, diabetes mellitus, or sex (all *p* > 0.1). Subgroup AUCs ranged from 0.726 to 0.842, with the highest values in the ≥75 years (0.832) and septic-shock (0.842) subgroups (Figure 3b; Table 2).

### Correlation of VAS score with disease-severity indicators

**Figure 4:**
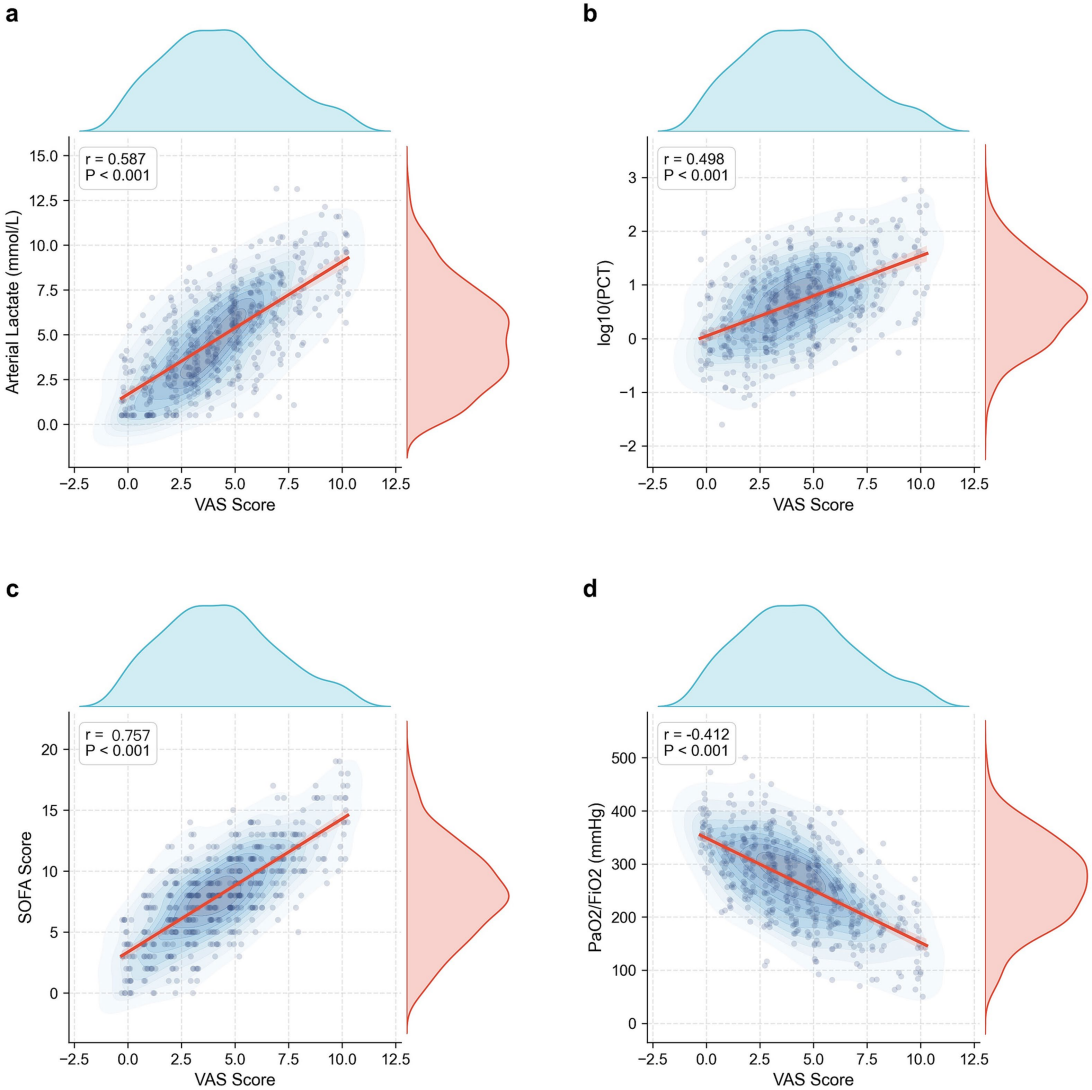
Correlation of the VAS score with disease-severity indicators. Scatter plots with marginal kernel-density curves; the red line is the linear regression fit. Spearman correlation: **(a)** Lactate (*r* = 0.587, *p* < 0.001); **(b)** log₁₀(PCT) (*r* = 0.498, *p* < 0.001); **(c)** SOFA score (*r* = 0.757, *p* < 0.001); **(d)** PaO₂/FiO₂ (*r* = −0.412, *p* < 0.001).

The VAS score correlated positively with lactate (*r* = 0.587), log₁₀(PCT) (*r* = 0.498), and SOFA score (*r* = 0.757), and negatively with PaO₂/FiO₂ (*r* = −0.412); all *p* < 0.001 (Figure 4).

### Predictive value for secondary outcomes

**Figure 5:**
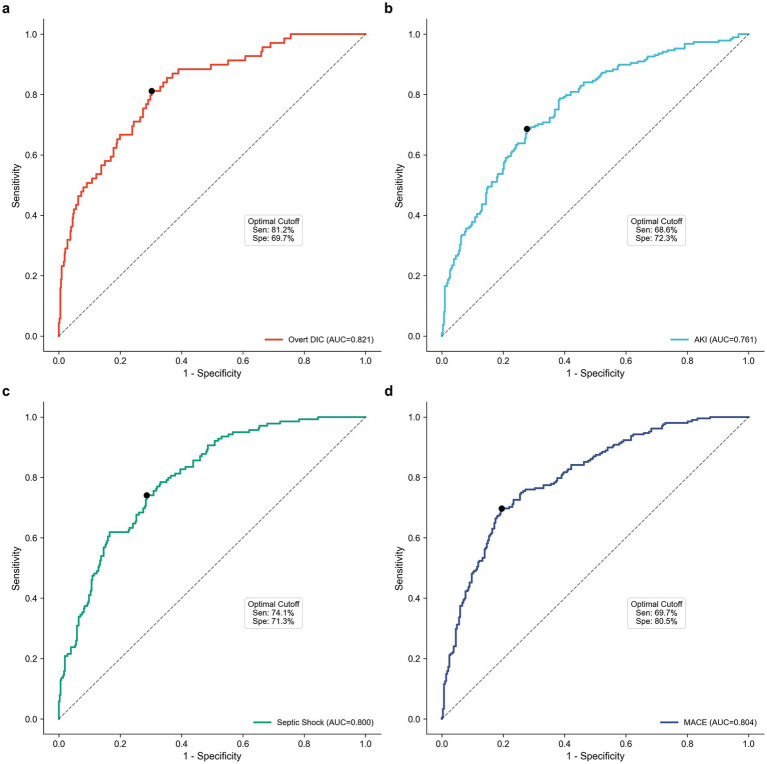
ROC curves for the VAS score predicting secondary outcomes; black dots indicate the optimal cut-off. **(a)** Overt DIC (AUC = 0.821; sensitivity 81.2%, specificity 69.7%); **(b)** AKI (AUC = 0.761; 68.6%/72.3%); **(c)** septic shock (AUC = 0.800; 74.1%/71.3%); **(d)** MACE composite endpoint (AUC = 0.804; 69.7%/80.5%).

The VAS score predicted all seven secondary outcomes with statistical significance (all *p* < 0.05). Ranked by AUC: overt DIC (0.821), MACE (0.804), septic shock (0.800), CRRT (0.798), invasive mechanical ventilation (0.768), AKI (0.761), and 30-day readmission (0.652) (Figure 5; Supplementary Table S1). Incidence of every secondary outcome was significantly higher in the VAS-high than VAS-low group (all *p* < 0.001 except 30-day readmission, *p* = 0.045), with the largest absolute differences for MACE (65.0% vs. 22.5%), AKI (55.8% vs. 25.3%), and invasive mechanical ventilation (41.7% vs. 14.9%) (Supplementary Figure S2). The MODS group peaked at VAS = 5, whereas the no-MODS group peaked at VAS = 2; the two distributions differed significantly (*χ*^2^ = 126.4, *p* < 0.001; Supplementary Figure S1). In the SIC-stratified analysis, the VAS score performed better in the SIC-positive subgroup (AUC = 0.806) than in the SIC-negative subgroup (AUC = 0.728, DeLong *p* = 0.047 vs. overall; Supplementary Table S3).

The nomogram built on age, VAS score, lactate, log₁₀(PCT), and CCI achieved a C-index of 0.842 (95% CI 0.804–0.880); the bootstrap-corrected C-index was 0.821, with calibration intercept of −0.018 and slope of 0.962 (predicted probability range 0.02–0.96) (Supplementary Figure S3).

## Discussion

In this single-centre retrospective cohort of 495 patients with sepsis, we systematically evaluated the VAS score for prediction of 28-day new-onset MODS. The principal findings are: (1) the VAS score (AUC = 0.777) significantly outperformed the SIC and qSOFA scores and was comparable to the more cumbersome SOFA score; (2) VAS ≥ 4 was an independent predictor of MODS (adjusted HR 3.42), with the strongest performance in elderly patients and those with septic shock; (3) addition of lactate, PCT, and NT-proBNP raised the AUC to 0.853; (4) dynamic ΔVAS deterioration was an independent predictor of organ-dysfunction progression; and (5) the VAS score also predicted overt DIC, AKI, septic shock, and the MACE composite endpoint with good discrimination. Together, these findings position the VAS score as a multidimensional, easy-to-deploy bedside tool with substantial clinical and translational potential.

The VAS score significantly outperformed the SIC score (*p* < 0.001) and was statistically equivalent to the SOFA score (*p* = 0.083). The clinical implication is that, although SOFA matches VAS in discrimination, its calculation requires complete dynamic data across six organ systems, which is rarely achievable at the point of early emergency-department evaluation (5, 18). By contrast, the VAS score adds only two readily available admission variables—age and vasopressor use—to the SIC framework, making it suitable for immediate bedside assessment. The APACHE II AUC point estimate (0.783) was slightly higher than that of VAS, with a small but statistically significant difference (*p* = 0.026); however, APACHE II requires aggregation of the worst physiological values over the first 24 h, introducing a time lag that limits its utility for early decision-making (6, 19). This advantage over the SIC score indicates that the age and vasopressor modules add independent and clinically meaningful information for MODS prediction, addressing well-described limitations of the original SIC score (9, 10). The superior performance of the SOFA score (AUC = 0.757) over the SIC score (AUC = 0.708, DeLong *p* < 0.001) likely reflects SOFA’s comprehensive assessment across six physiological systems, whereas the SIC score is anchored on the coagulation axis combined with a binary SOFA component, thereby capturing a narrower dimension of organ dysfunction and yielding lower discrimination for a multi-system endpoint such as MODS. Compared with SOFA-2 (AUC = 0.791), which incorporates 24-h dynamic information and is widely used as a more responsive organ-dysfunction indicator, the VAS score achieved comparable discrimination (*p* = 0.412) while requiring only a single bedside assessment at admission, further supporting its practical advantage for early risk stratification.

Sequential incorporation of biomarkers into the VAS framework yielded statistically significant NRI and IDI improvements at each step. The full biomarker model (VAS + lactate + PCT + NT-proBNP, AUC = 0.853) substantially outperformed VAS alone (NRI = 0.124, *p* = 0.012; IDI = 0.086, *p* = 0.021). Mechanistically, elevated lactate reflects tissue hypoperfusion and mitochondrial dysfunction, and is among the most sensitive early biochemical indicators of organ injury, with established associations with sepsis MODS and mortality (20, 21); PCT, a highly specific biomarker of bacterial infection and systemic inflammation, correlates with sepsis severity and supports early prognostication (22, 23); and NT-proBNP elevation indicates septic cardiomyopathy and elevated cardiac filling pressures, which contribute to MODS amplification (24, 25). These three biomarkers provide complementary metabolic, inflammatory, and cardiac information not fully captured by the VAS score alone. DCA further showed that, across the clinically relevant threshold-probability range of 15–55%, the VAS + lactate combination delivered greater net benefit than VAS alone, supporting practical decision optimisation in patients of intermediate risk (26).

Subgroup analysis revealed clinically meaningful heterogeneity. The age interaction (P-int = 0.024) showed the highest HR (4.86) and AUC (0.832) in the ≥75-years subgroup, indicating that the prognostic value of the VAS score is amplified with advancing age. This is consistent with the unique vulnerability of elderly patients: immunosenescence, low-grade chronic inflammation (“inflammaging”), and reduced organ reserve predispose this group to dysregulated inflammatory cascades and organ failure following infection (12, 13). Given the global rise in elderly ICU admissions and their disproportionately high MODS and mortality rates (27, 28), the targeted incorporation of age in the VAS framework addresses an increasingly pressing clinical need. The septic-shock subgroup likewise showed a stronger HR (4.62 vs. 2.84 in non-shock; P-int = 0.038), suggesting that the vasopressor module contributes most strongly in patients with the greatest circulatory burden. Recent evidence indicates dose-dependent associations between catecholamine exposure and adverse organ-injury and survival outcomes (14, 15), and the pathobiology of vascular smooth muscle cell dysfunction has been increasingly recognised as a central driver of sepsis-induced vasoplegia and downstream organ injury (29), reinforcing the rationale for explicit integration of vasopressor status into risk stratification.

ΔVAS analysis demonstrated that VAS deterioration over 48 h independently predicted MODS (HR = 4.78, *p* < 0.001), exceeding the prognostic value of single-time-point baseline VAS (univariable HR = 1.42 per point). This aligns with the inherently dynamic pathophysiology of sepsis-related organ dysfunction, in which infection–inflammation–coagulation–microcirculation interactions evolve in time-dependent fashion (30). Trajectory-based monitoring strategies have shown superior prognostic discrimination over single-point assessment in multiple critical-illness studies, providing a more responsive framework for real-time decision-making (31). In practice, ΔVAS could function as a quantitative measure of treatment response: continuing improvement aligns with favourable prognosis and supports resource de-escalation, whereas deterioration may prompt timely escalation of organ support.

The VAS score predicted all seven secondary outcomes with statistical significance (AUC range 0.652–0.821), with the strongest performance for overt DIC (AUC = 0.821) and septic shock (AUC = 0.800). This broad predictive capacity is consistent with the multidimensional design of the score: SIC-related coagulation activation contributes to AKI, hepatic injury, and myocardial injury (8, 11), while vasopressor exposure relates directly to hypoperfusion-driven renal, cardiac, and pulmonary injury (14, 32). The relatively lower performance for 30-day readmission (AUC = 0.652) likely reflects the dependence of readmission on post-discharge social support, comorbidity management, and chronic health status rather than acute disease severity (33), illustrating an inherent boundary for any acute-phase prognostic score.

Most existing composite tools for sepsis MODS prediction extend the SOFA score, with few systematically integrating coagulation, age, and circulation in a single framework. Compared with previous SIC-validation studies, our findings not only confirm the independent predictive value of coagulation assessment but also demonstrate systematic performance gains achieved by adding age and vasopressor modules. Notably, VAS performance was more stable in SIC-positive (AUC = 0.806) than in SIC-negative patients (AUC = 0.728), suggesting greater applicability in patients with substantial coagulopathy burden (9, 10). The internally validated nomogram (C-index = 0.842; bootstrap-corrected C-index = 0.821) provides a practical instrument for individualised risk quantification that has not been systematically reported in previous sepsis-prediction modelling studies (16, 17). It should be emphasised that the nomogram is intended as a complementary refinement tool rather than a replacement for the VAS score: the VAS score itself remains the simple bedside instrument for immediate risk stratification, whereas the nomogram—which additionally incorporates log₁₀(PCT) and CCI—is best suited to settings where these variables are already routinely available in the electronic health record or where higher-precision individualised risk estimation is required (e.g., resource allocation, research stratification, or risk communication), and can be deployed via a freely available online calculator to obviate manual computation.

Our study has several limitations. First, the single-centre retrospective design introduces potential selection and information biases, and external generalisability requires multicentre prospective validation. Second, the PT-ratio component depends on local control values, which may limit cross-centre comparability. Third, the vasopressor module is binary and does not capture dosage, which could be refined by adopting the Vasoactive-Inotropic Score (VIS). Fourth, MODS adjudication relied on retrospective reconstruction of daily SOFA scores, which is sensitive to documentation completeness. Finally, although the events-per-variable ratio of 21.1 supports model robustness, the sample size (n = 495) is moderate, and larger validation cohorts are warranted.

## Conclusion

The VAS score is a parsimonious multidimensional bedside tool that independently predicts 28-day new-onset MODS in patients with sepsis, significantly outperforming the SIC score and matching the SOFA score with simpler computation. A cut-off of VAS ≥ 4 provides a clinically actionable threshold for early identification of high-risk patients; combination with circulating biomarkers substantially improves discrimination; and dynamic ΔVAS monitoring offers a quantitative measure of organ-function trajectories. The accompanying nomogram is intended as an optional refinement tool for individualised risk quantification rather than a replacement for the bedside VAS score. Multicentre prospective studies are needed for external validation and for evaluating the feasibility of integrating the VAS score into clinical decision-support systems.

## Data Availability

The raw data supporting the conclusions of this article will be made available by the authors, without undue reservation.

## Glossary

AKI: Acute Kidney Injury
APACHE II: Acute Physiology and Chronic Health Evaluation II
AUC: Area under the Receiver Operating Characteristic Curve
CCI: Charlson Comorbidity Index
CI: Confidence Interval
CKD: Chronic Kidney Disease
CRP: C-Reactive Protein
CRRT: Continuous Renal Replacement Therapy
DCA: Decision Curve Analysis
DIC: Disseminated Intravascular Coagulation
HR: Hazard Ratio
ICU: Intensive Care Unit
IDI: Integrated Discrimination Improvement
IQR: Interquartile Range
ISTH: International Society on Thrombosis and Haemostasis
KDIGO: Kidney Disease: Improving Global Outcomes
LR: likelihood Ratio
MACE: Major Adverse Clinical Events
MODS: Multiple Organ dysfunction Syndrome
NPV: Negative Predictive Value
NRI: Net Reclassification Improvement
NT-proBNP: N-Terminal pro-B-type Natriuretic Peptide
PaO₂/FiO₂: Ratio of Arterial Oxygen Partial Pressure to Fractional Inspired Oxygen
PCT: Procalcitonin
PPV: Positive Predictive Value
PT: Prothrombin Time
qSOFA: Quick Sequential Organ Failure Assessment
ROC: Receiver Operating Characteristic
SIC: Sepsis-Induced Coagulopathy
SOFA: Sequential Organ Failure Assessment
VAS: Vasoactive-Age adjusted SIC score
VIS: Vasoactive-Inotropic Score. SOFA-2, 24-h worst SOFA Score
