# Supplementary material for: The vasoactive-age adjusted sepsis-induced coagulopathy score predicts 28-day new-onset multiple organ dysfunction syndrome in patients with sepsis: a single-centre retrospective cohort study
Source: Front Med (Lausanne). 2026 Jul 6;13:1874204. doi: 10.3389/fmed.2026.1874204 (PMC13381841; doi:10.3389/fmed.2026.1874204)
Supplement: Supplementary file 1 [file Supplementary_file_1.docx]

**
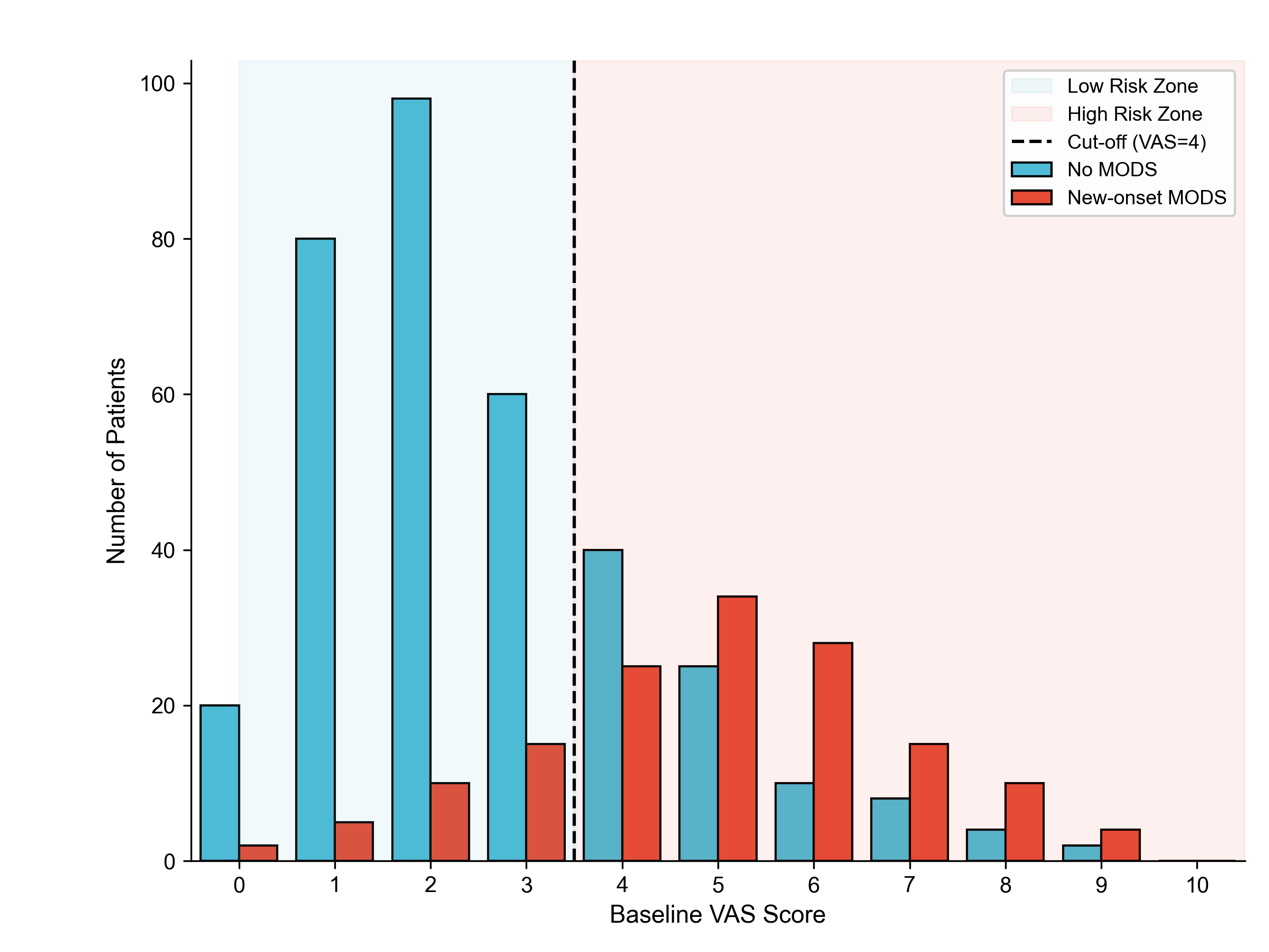
**

**Supplementary Figure S1.** Histogram of baseline VAS-score distribution. Light-blue bars: no-MODS group; dark-red bars: new-onset MODS group. Vertical dashed line at VAS=4 marks the optimal cut-off. The no-MODS distribution peaks at VAS=2 (n=98), the MODS distribution at VAS=5 (n=34); χ²=126.4, P<0.001.

**
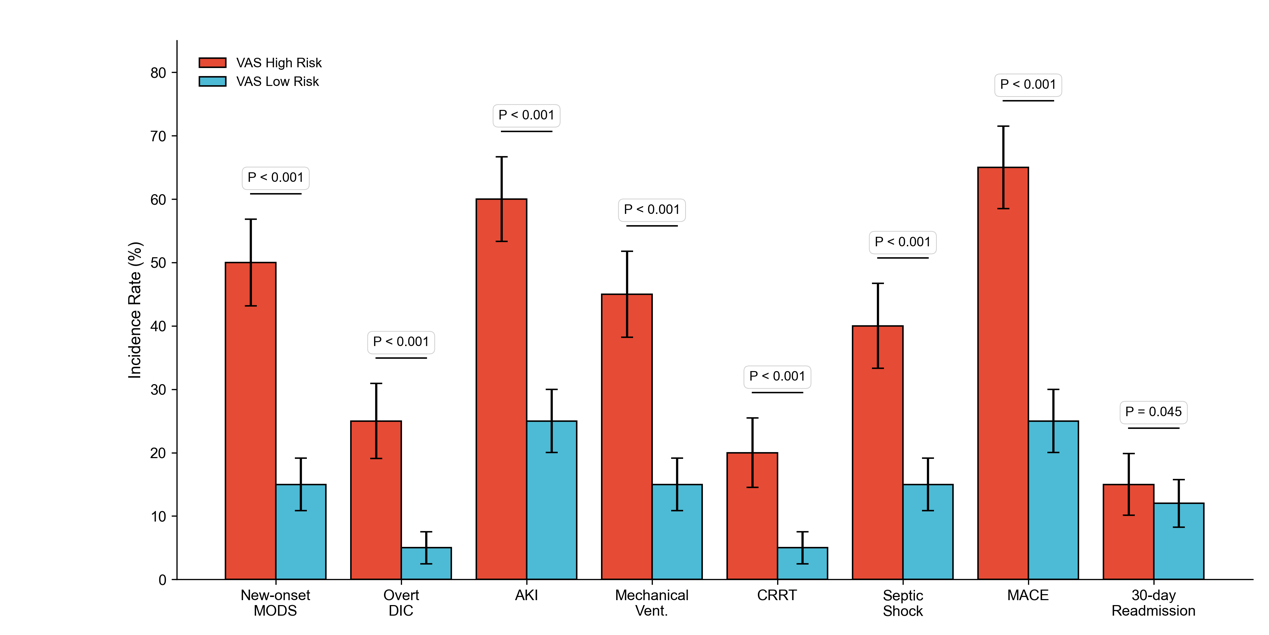
**

**Supplementary Figure S2.** Incidence of clinical outcomes in VAS-high (n=206, red) versus VAS-low (n=289, light blue) groups. Error bars represent 95% CIs; P values from χ² tests. All outcomes differed significantly (P<0.001 except 30-day readmission, P=0.045).


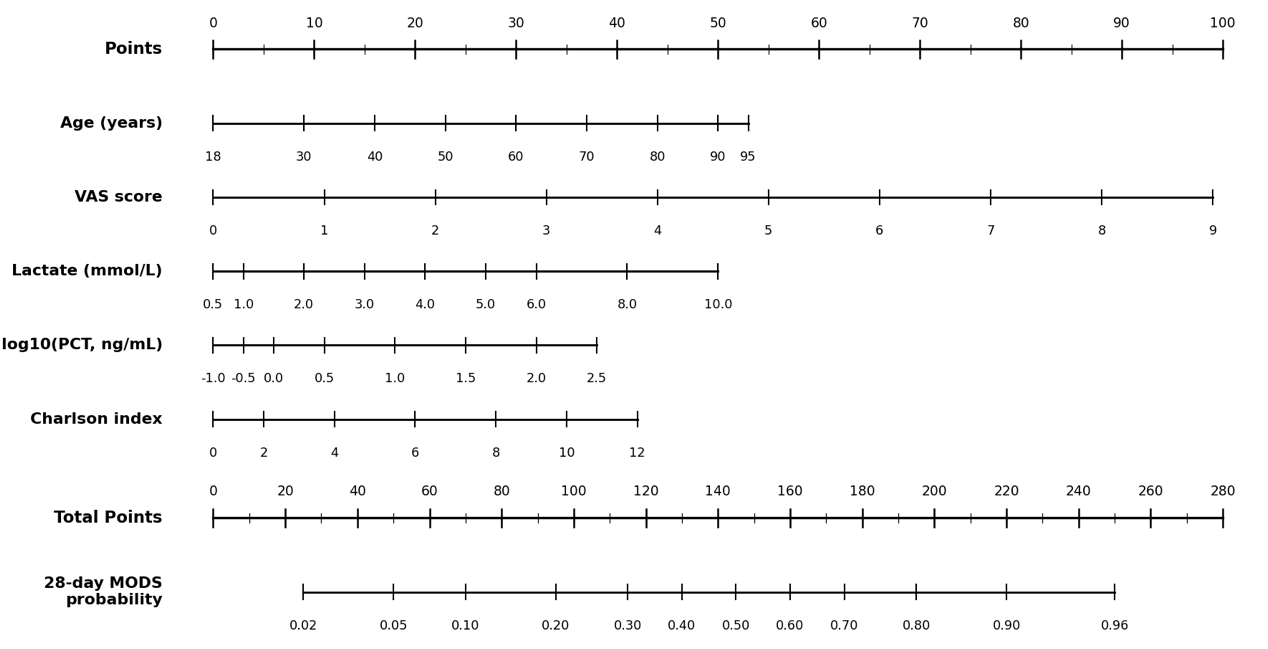


**Supplementary Figure S3.** Nomogram for individualised prediction of 28-day new-onset MODS. Variables (top to bottom): age, VAS score, lactate, log₁₀(PCT), and CCI. Total points map to predicted probability of MODS (range 0.02–0.96). C-index=0.842 (95% CI 0.804–0.880); bootstrap-corrected C-index=0.821; calibration intercept –0.018, slope 0.962.


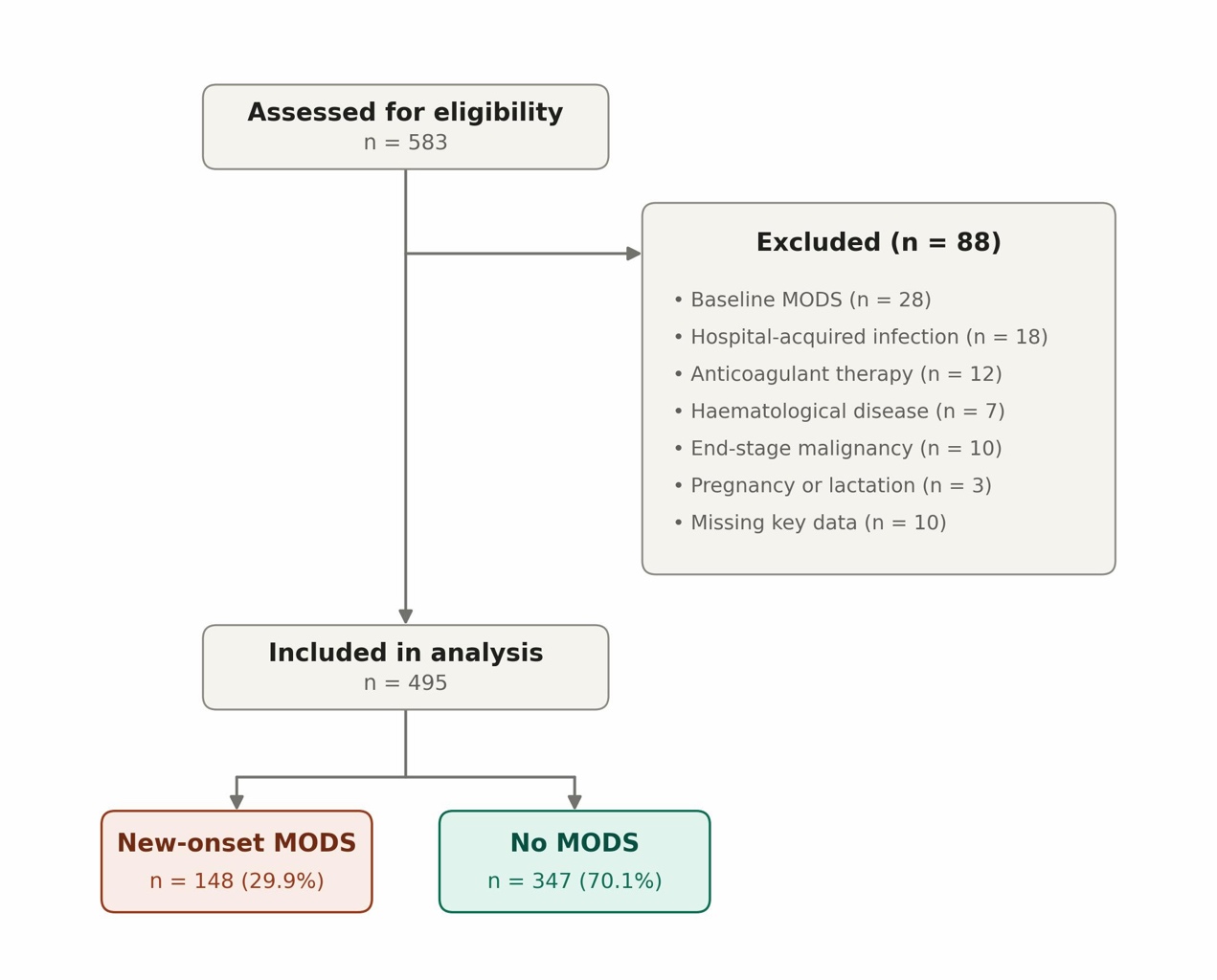


**Supplementary Figure S4.** CONSORT-style flow diagram of patient selection.
