# Supplementary material for: The vasoactive-age adjusted sepsis-induced coagulopathy score predicts 28-day new-onset multiple organ dysfunction syndrome in patients with sepsis: a single-centre retrospective cohort study
Source: Front Med (Lausanne). 2026 Jul 6;13:1874204. doi: 10.3389/fmed.2026.1874204 (PMC13381841; doi:10.3389/fmed.2026.1874204)
Supplement: Supplementary file 2 [file Table_1.docx]

**Supplementary Tables**

**Supplementary Table S1. Predictive performance of VAS score for secondary outcomes**

| **Outcome** | **Events, n** | **Incidence, %** | **AUC (95% CI)** | **Optimal cutoff** | **Sensitivity, %** | **Specificity, %** | **P value** |
| --- | --- | --- | --- | --- | --- | --- | --- |
| Overt DIC (ISTH 2001) | 69 | 13.9 | 0.821 (0.758–0.866) | ≥4 | 81.2 | 69.7 | <0.001 |
| AKI (KDIGO 2012) | 188 | 38.0 | 0.761 (0.694–0.792) | ≥3 | 68.6 | 72.3 | <0.001 |
| New invasive ventilation | 129 | 26.1 | 0.768 (0.722–0.814) | ≥4 | 72.1 | 70.5 | <0.001 |
| New CRRT initiation | 45 | 9.1 | 0.798 (0.728–0.868) | ≥5 | 73.3 | 76.2 | <0.001 |
| Septic shock | 139 | 28.1 | 0.800 (0.781–0.825) | ≥4 | 74.1 | 71.3 | <0.001 |
| MACE composite endpoint | 208 | 42.0 | 0.804 (0.762–0.842) | ≥4 | 69.7 | 80.5 | <0.001 |
| 30-day readmission | 64 | 12.9 | 0.652 (0.588–0.716) | ≥3 | 60.9 | 64.2 | 0.018 |

DIC, disseminated intravascular coagulation (ISTH 2001 overt DIC criteria, score ≥5); AKI, acute kidney injury (KDIGO 2012 criteria); CRRT, continuous renal replacement therapy; MACE composite endpoint defined as the occurrence of any one of: new-onset MODS, new invasive ventilation, new CRRT initiation, new overt DIC, or escalation from general ward to ICU. AUC, area under the receiver operating characteristic curve; CI, confidence interval.

**Supplementary Table S2. Association between dynamic ΔVAS score (T48−T0) and 28-day new-onset MODS**

| **ΔVAS group** | **n** | **MODS events, n** | **Incidence, %** | **HR (95% CI)ᵃ** | **P value** |
| --- | --- | --- | --- | --- | --- |
| Improved (ΔVAS <0) | 142 | 18 | 12.7 | 1.00 (ref.) | — |
| Unchanged (ΔVAS =0) | 226 | 64 | 28.3 | 2.31 (1.37–3.89) | 0.002 |
| Worsened (ΔVAS >0) | 127 | 66 | 52.0 | 4.78 (2.83–8.07) | <0.001 |
| Trend test | — | — | — | — | <0.001 |

ᵃ Multivariable Cox proportional hazards regression adjusted for age, Charlson Comorbidity Index, source of infection, time to antibiotic initiation, and baseline VAS score. Baseline VAS was included to ensure that the estimated effect of ΔVAS represents the independent prognostic value of the dynamic trajectory beyond the starting score. ΔVAS defined as VAS score at 48 h minus VAS score at admission. MODS, multiple organ dysfunction syndrome; HR, hazard ratio; CI, confidence interval.

**Supplementary Table S3. Predictive value of VAS score in SIC-positive vs. SIC-negative subgroups**

| **Subgroup** | **n** | **MODS events, n** | **AUC (95% CI)** | **Optimal cutoff** | **Sensitivity, %** | **Specificity, %** | **DeLong P valueᵃ** |
| --- | --- | --- | --- | --- | --- | --- | --- |
| SIC-negative (SIC <4) | 298 | 56 | 0.728 (0.658–0.798) | ≥3 | 67.9 | 68.6 | 0.047 |
| SIC-positive (SIC ≥4) | 197 | 92 | 0.806 (0.742–0.870) | ≥5 | 70.7 | 73.3 | 0.756 |
| Overall cohort | 495 | 148 | 0.777 (0.755–0.841) | ≥4 | 76.4 | 73.2 | — |

ᵃ DeLong method comparing each subgroup AUC with the overall cohort AUC. The AUC in SIC-negative patients (0.728) was significantly lower than the overall AUC (0.777; P=0.047), whereas the AUC in SIC-positive patients (0.806) did not differ significantly (P=0.756), indicating stable predictive value in the SIC-positive stratum. SIC, sepsis-induced coagulopathy; AUC, area under the ROC curve; CI, confidence interval.

**Supplementary Table S4. Combined SIC×VAS stratification and 28-day new-onset MODS incidence**

| **Subgroup** | **n** | **%** | **MODS events, n** | **Incidence, %** | **Adjusted HR (95% CI)ᵃ** | **P value** |
| --- | --- | --- | --- | --- | --- | --- |
| SIC-negative + VAS low-risk (ref.) | 232 | 46.9 | 21 | 9.1 | 1.00 (ref.) | — |
| SIC-negative + VAS high-risk | 66 | 13.3 | 35 | 53.0 | 6.85 (4.02–11.66) | <0.001 |
| SIC-positive + VAS low-risk | 57 | 11.5 | 14 | 24.6 | 2.84 (1.43–5.64) | 0.003 |
| SIC-positive + VAS high-risk | 140 | 28.3 | 78 | 55.7 | 7.42 (4.55–12.10) | <0.001 |
| Trend test | — | — | — | — | — | <0.001 |

ᵃ Multivariable Cox proportional hazards regression adjusted for age, Charlson comorbidity index, source of infection, time to antibiotic initiation, and 6-h fluid resuscitation volume. In SIC-negative patients, VAS high-risk was associated with a ~6-fold higher MODS incidence vs. VAS low-risk (53.0% vs. 9.1%), indicating that the vasopressor/age-adjustment modules of VAS provide prognostic information independent of coagulation status. SIC, sepsis-induced coagulopathy; HR, hazard ratio; CI, confidence interval.

**Supplementary Table S5.** **Proportion of missing values for each key variable in the analytic cohort**

| **Variable** | **Missing, n** | **Missing, %** |
| --- | --- | --- |
| PT ratio | 2 | 0.4 |
| Platelet count | 0 | 0 |
| Arterial lactate | 6 | 1.2 |
| Procalcitonin (PCT) | 12 | 2.4 |
| NT-proBNP | 23 | 4.6 |
| C-reactive protein (CRP) | 9 | 1.8 |
| Serum creatinine | 0 | 0 |
| PaO₂/FiO₂ | 10 | 2.0 |
| 6-h fluid resuscitation volume | 7 | 1.4 |
| Time to first antibiotic dose | 8 | 1.6 |

Missing data were <5% for every variable. Missing continuous values were addressed by multiple imputation by chained equations (MICE, m = 5) under the missing-at-random assumption; categorical variables had no missing values. PT, prothrombin time; PCT, procalcitonin; NT-proBNP, N-terminal pro-B-type natriuretic peptide; CRP, C-reactive protein; PaO₂/FiO₂, ratio of arterial oxygen partial pressure to fractional inspired oxygen.
